# Variability of Suitable Habitat of Western Winter-Spring Cohort for Neon Flying Squid in the Northwest Pacific under Anomalous Environments

**DOI:** 10.1371/journal.pone.0122997

**Published:** 2015-04-29

**Authors:** Wei Yu, Xinjun Chen, Qian Yi, Yong Chen, Yang Zhang

**Affiliations:** 1 College of Marine Sciences, Shanghai Ocean University, Shanghai, 201306, China; 2 National Engineering Research Center for Oceanic Fisheries, Shanghai Ocean University, Shanghai, 201306, China; 3 Key Laboratory of Sustainable Exploitation of Oceanic Fisheries Resources, Ministry of Education, Shanghai Ocean University, Shanghai, 201306, China; 4 School of Marine Sciences, University of Maine, Orono, Maine, 04469, United States of America; 5 Collaborative Innovation Center for Distant-water Fisheries, Shanghai, 201306, China

## Abstract

We developed a habitat suitability index (HSI) model to evaluate the variability of suitable habitat for neon flying squid (*Ommastrephes bartramii*) under anomalous environments in the Northwest Pacific Ocean. Commercial fisheries data from the Chinese squid-jigging vessels on the traditional fishing ground bounded by 35°-45°N and 150°-175°E from July to November during 1998-2009 were used for analyses, as well as the environmental variables including sea surface temperature (SST), chlorophyll-a (Chl-a) concentration, sea surface height anomaly (SSHA) and sea surface salinity (SSS). Two empirical HSI models (arithmetic mean model, AMM; geometric mean model, GMM) were established according to the frequency distribution of fishing efforts. The AMM model was found to perform better than the GMM model. The AMM-based HSI model was further validated by the fishery and environmental data in 2010. The predicted HSI values in 1998 (high catch), 2008 (average catch) and 2009 (low catch) indicated that the squid habitat quality was strongly associated with the ENSO-induced variability in the oceanic conditions on the fishing ground. The La Niña events in 1998 tended to yield warm SST and favorable range of Chl-a concentration and SSHA, resulting in high-quality habitats for *O*. *bartramii*. While the fishing ground in the El Niño year of 2009 experienced anomalous cool waters and unfavorable range of Chl-a concentration and SSHA, leading to relatively low-quality squid habitats. Our findings suggest that the La Niña event in 1998 tended to result in more favorable habitats for *O*. *bartramii* in the Northwest Pacific with the gravity centers of fishing efforts falling within the defined suitable habitat and yielding high squid catch; whereas the El Niño event in 2009 yielded less favorable habitat areas with the fishing effort distribution mismatching the suitable habitat and a dramatic decline of the catch of *O*. *bartramii*. This study might provide some potentially valuable insights into exploring the relationship between the underlying squid habitat and the inter-annual environmental change.

## Introduction

**Fig 1:**
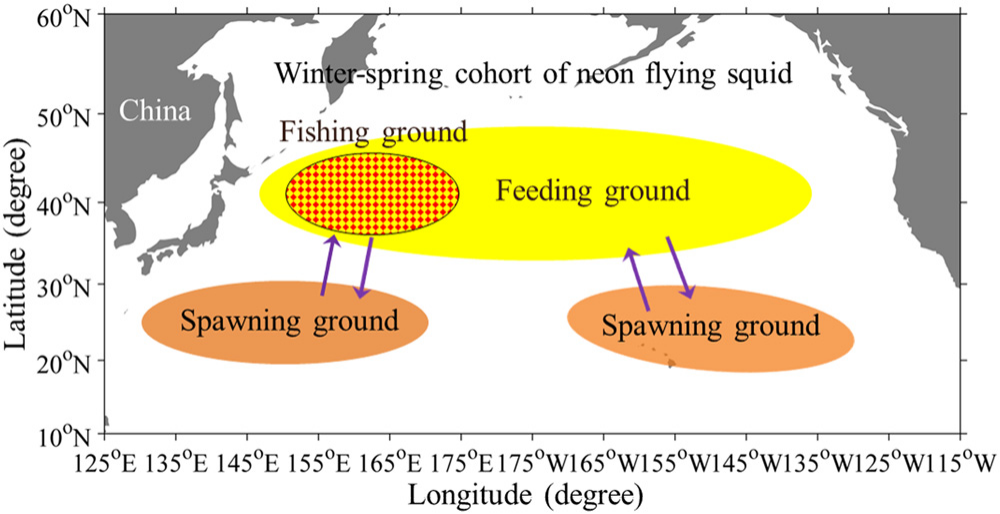
The migration pattern of winter-spring cohort of *Ommastrephes bartramii*. The brown and yellow areas show the spawning and feeding grounds, respectively, in the North Pacific. The red area is the traditional fishing ground for the Chinese squid-jigging vessels in the Northwest Pacific Ocean.

*Ommastrephes bartramii*, commonly known as neon flying squid, is the most abundant and economically important oceanic squid in the family Ommastrephidae widely distributed in subtropical and temperate waters of the world’s ocean [1–3]. The North Pacific population of neon flying squid is mainly distributed between 20°N and 50°N, and comprises of two spawning cohorts: an autumn cohort and a winter-spring cohort, both of which have a 1-year lifespan [4,5]. The western stock of winter-spring cohort of neon flying squid annually undertakes round-trip migration from subtropical waters into the subarctic domain [6,7], where the warm Kuroshio Current and the cold Oyashio Current meet, sustaining the most productive traditional fishing ground between 35°-45°N and 150°-175°E for the Chinese squid-jigging fishery (Fig 1). The Japanese jigging vessels first commercially exploited this species in 1974, while Chinese Mainland started to survey the resource by using squid-jigging vessels in 1993 in the Northwest Pacific, and subsequently started a large-scale commercial production after 1994. Annual catch of *O*. *bartramii* in China was maintained at 80000–100000 t, of which the western winter-spring cohort of neon flying squid was the main fishing target by the Chinese squid-jigging vessels [8].

As short-lived ecological opportunists, the abundance and distribution of cephalopod stocks are extremely sensitive to the multi-scale environmental conditions [9,10]. Previous results indicated that the distribution of fishing ground and abundance of *O*. *bartramii* were closely related to meanderings of the Kuroshio and Oyashio Currents [11], the occurrence of El Niño and La Niña events [12], variations in the sea surface water temperature [13], vertical temperature structure [14] and chlorophyll-a (Chl-a) concentration [15] on the fishing ground. The large-scale environmental variability might lead to the significant fluctuations in the spatio-temporal distribution of fishing ground and squid stock level, especially when the anomalous environmental conditions occurred [16–18]. For example, Nigmatullin et al. [10] suggested that El Niño phenomenon would result in a reduced population size of *Dosidicus gigas* and subsequently a large decline in fishery yields. Waluda et al. [19] examined the abundance of *D*. *gigas* and fleet distributions in Peruvian waters during three different environmental variations with intermediate (1994), La Niña (1996) and El Niño (1997) conditions, and found that the cool or warm temperature anomalies caused by La Niña and El Niño events would result in low catches. Xu et al. [20] concluded that El Niño and La Niña events played an important role in regulating the distribution of fishing ground of *D*. *gigas*, and the main fishing ground during the La Niña years moved to further northward for 1–2°N and the average SST of the fishing grounds decreased by 2°C compared to that in the El Niño years. For *O*. *bartramii*, Chen et al. [12] suggested that the large-scale environmental change had great influences on the oceanographic conditions on the spawning and fishing grounds, resulting in recruitment variability and spatio-temporal changes of fishing grounds of the western stock under various anomalous environments. They concluded that the La Niña events tended to provide unfavorable environmental conditions on the spawning ground, the squid recruitment would reduce, and the fishing ground might move northward, whereas in the El Niño years the habitat on the spawning ground would favorable to squid recruitment, the fishing ground tended to shift southward [12].

Habitat suitability index (HSI) modeling combined with geographic information system (GIS) has been extensively used for improving the management and exploitation of fisheries resources [21,22]. Habitat suitability index model was initially proposed to describe the quality of wildlife habitat by the U.S. Geological Survey National Wetlands Research Center and Fish and Wildlife Department in the early 1980s, but later the HSI modeling method was applied to ecological restoration research [23,24] and exploration of fishing ground of pelagic species [25,26]. There have been efforts to evaluate the relationship between the optimal habitat of pelagic species and the environmental variability using the HSI approach. For example, Chang et al. [27] developed a HSI model to evaluate the influences of oceanographic conditions and climate change on the habitat suitability of swordfish, *Xiphias gladius*, in the equatorial Atlantic Ocean. They found the spatial shifts in the optimal habitat were greatly related to the Niño-Southern Oscillation and/or Northern Atlantic Oscillation. The HSI modelling has been utilized for the purpose of defining the suitable range of environmental variables and identifying the optimal fishing grounds for *O*. *bartramii* [28,29]. However, limit efforts are made to examine the linkage between the spatio-temporal dynamics of suitable habitat and the environmental events, and we have limited understanding of how the suitable habitat of the western stock of winter-spring cohort may respond to the inter-annual oceanographic variability, especially to the anomalous environments?

Large fluctuations were observed in the oceanic conditions and the production of western winter-spring cohort of neon flying squid in the Northwest Pacific in recent years. Previous findings also implied that the variability in the squid abundance was probably driven by the dynamics of suitable habitat areas on the fishing ground which was linked to the large-scale environmental change (e.g., La Niña and El Niño events) [12,30]. Thus, it is important to extend the research to explore in details how the anomalous environmental conditions impacted the squid habitat in three typical years with the La Niña, average and El Niño conditions, respectively, using HSI modeling. In this study, we assumed that the HSI values varied under different environmental conditions, and the optimal habitat was closely correlated with fishing effort. Extensive evidence showed that the distribution and availability of *O*. *bartramii* to fisheries can be strongly influenced by environmental variables such as SST, SSS, sea surface height anomaly (SSHA) and Chl-a concentration [31–33]. Therefore, we used the relevant environmental variables above-mentioned to develop a HSI model for *O*. *bartramii*. The goal of this study are to identify the relationship between the squid habitat and environmental variables on the fishing ground and to evaluate the impacts of La Niña and El Niño events on the spatio-temporal changes of the squid preferred habitat.

## Materials and Methods

### Fishery data

The squid fishery logbook data during July to November over 1998–2010 were available from the Chinese Squid-jigging Technology Group of Shanghai Ocean University. The logbook information included data on daily catch (t), fishing effort (days fished), fishing dates (year and month) and locations (longitude and latitude), at the resolution of 1° latitude by 1° longitude, on the traditional fishing ground for Chinese squid-jigging vessels bounded by 35°-45°N and 150°-175°E [8,12].

The Chinese squid-jigging vessels were equipped with almost identical engine and fishing power, the vessels were similar in size and nighttime fishing operation and protocol. There were no bycatch species in the Chinese squid fishery [12,30]. Therefore, catch per unit effort (CPUE) tended to be a reliable indicator of squid abundance [8]. The nominal CPUE in the fishing unit of 1°×1° was calculated as follows:

$$CPUE_{ymij}=\frac{C_{ymij}}{F_{ymij}}$$ (1)

where *CPUE*_*ymij*_,*C*_*ymij*_ and *F*_*ymij*_ were the nominal CPUE (tons/day, t/d), the sum of catch (tons, t) for all the fishing vessels within a fishing grid and the sum of all fishing days of all fishing vessels within a fishing grid, respectively, at longitude *i*, latitude *j* in month *m* and year *y*. The fishing efforts for each month were calculated as the total integer fishing vessel days within a fishing grid of 1°×1° [28].

### Environmental data

Four environmental variables from the remote sensing data including SST, Chl-a concentration, SSHA and SSS were considered based on their shown importance to the distribution and abundance of *O*. *bartramii* [28,34]. The monthly SST data were obtained from the NOAA High-resolution Blended Analysis (http://apdrc.soest.hawaii.edu/data) with a resolution of 1.0°×1.0°. The monthly SSS data were obtained from the IRI/LDEO Climate Data Library (http://iridl.ldeo.columbia.edu), the spatial resolution was 1°×1/3°. The monthly SSHA and Chl-a concentration data were obtained from the Ocean-Watch Data Library (http://oceanwatch.pifsc.noaa.gov/las/servlets/dataset), with a resolution of 0.25°×0.25° and 0.1°×0.1°, respectively. All the environmental variables were averaged by 1°×1° for each month to match the spatio-temporal resolution of fishery data. The sea surface temperature anomaly (SSTA) data for the Niño 3.4 region were sourced from the NOAA Climate Prediction Center (http://www.cpc.ncep.noaa.gov/).

### HSI modelling

Fishing effort and CPUE (same definitions as our study) were generally considered as reliable indices of squid occurrence and abundance, respectively [35], for developing the HSI models [36,37]. However, the fishing effort tended to be better in constructing the HSI model compare to the CPUE-based HSI model [28]. Therefore, we used the fishing effort in relation to the environmental variables to calculate the suitability index (SI) for *O*. *bartramii* in this study. According to the frequency distribution of the fishing effort on the environmental variables, the SI for each environmental variable in each month (from July to November) was expressed as follows [38]:

$$SI=\frac{Effort_{i}}{Effort_{i,\max}}$$ (2)

where *Effort*_*i*_ was the total fishing efforts in the *i*th interval of the environmental variable range; *Effort*_*i*,max_ was the maximum total fishing efforts in the *i*th interval of the environmental variable range. We considered the fishing location with the lowest cumulative fishing efforts as the poorest habitat (SI = 0), representing the most unfavorable environmental condition, whereas the fishing location with the maximum cumulative fishing efforts was considered as the most suitable habitat (SI = 1), implying the most favorable environmental condition [39]. The SI values and each class interval value of the four environmental variables were then included for constructing the SI models. The relationship between the SI and each environmental variable was quantified by the following equations [40]:

$$SI_{SST}=a\exp\left[b{\left(X_{SST}-c\right)}^{2}\right]$$ (3)

$$SI_{Chl-a}=a\exp\left[b{\left(X_{Chl-a}-c\right)}^{2}\right]$$ (4)

$$SI_{SSHA}=a\exp\left[b{\left(X_{SSHA}-c\right)}^{2}\right]$$ (5)

$$SI_{SSS}=a\exp\left[b{\left(X_{SSS}-c\right)}^{2}\right]\;$$ (6)

where a, b and c were the model parameters to be estimated; *X*_*sst*_, *X*_*Chl-a*_, *X*_*SSHA*_ and *X*_*sss*_ were the values of each environmental variable. The range of each environmental variable with SI values higher than 0.6 was regarded as the suitable range for squid stocks [27,28]. The construction and statistical analysis for all SI models were implemented in Data Processing System (DPS) [40].

The SI models of the four environmental variables were then combined into the HSI models. Two empirical HSI models, the arithmetic mean model (AMM) [27,40] and the geometric mean model (GMM) [41,42] were extensively employed to estimate habitat suitability. In this study, the AMM and GMM models were established as:

$$HSI_{AMM}=\frac{1}{n}\overset{n}{\underset{i=1}{\sum }}\left(SI_{SST}+SI_{Chl-a}+SI_{SSHA}+SI_{SSS}\right)$$ (7)

$$HSI_{GMM}=\sqrt[{n}]{\overset{n}{\underset{i=1}{\prod }}\left(S_{SST},SI_{Chl-a},SI_{SSHA},SI_{SSS}\right)}$$ (8)

where *SI*_*SST*_, *SI*_*Chl-a*_, *SI*_*SSHA*_ and *SI*_*SSS*_ were SI values for each environmental variable; *n* was the number of environmental variables included in the HSI model. The HSI values were within the range between 0 and 1.

### HSI model selection and validation

**Fig 2:**
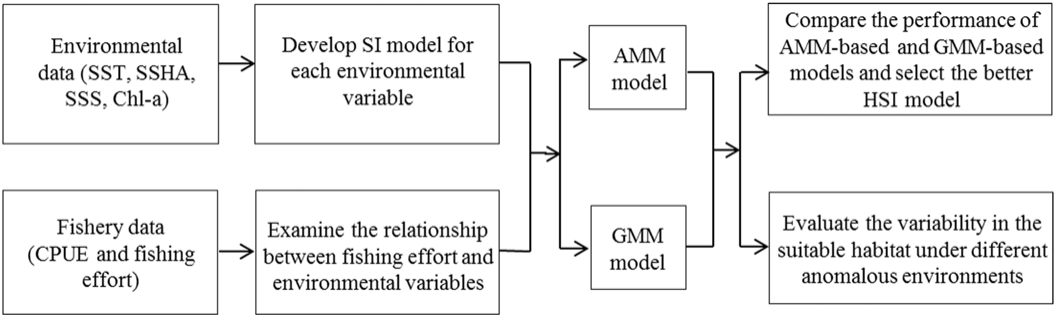
Schematic diagram to show the procedure of developing habitat suitability index (HSI) model for *Ommastrephes bartramii* in the Northwest Pacific. The HSI model contains four environmental variables, including sea surface temperature (SST), sea surface height anomaly (SSHA), sea surface salinity (SSS) and chlorophyll-a (Chl-a) concentration, respectively.

Fishery and environmental data during 1998–2009 were applied to establish the HSI model. The average CPUE and the percentage of fishing effort were evaluated in each HSI stratum (i.e. HSI = [0.0 0.2]; [0.2 0.4]; [0.4 0.6]; [0.6 0.8]; and [0.8 1.0]). In theory, most of the fishing efforts tended to be distributed in the waters with high SI values, little effort occur in the poor habitat, and the percentage of fishing effort should increase with the HSI values, but the average CPUE in each range of HSI values might fluctuate [43]. Based on this theory, we compared the performance of AMM and GMM models to choose a more suitable model to predict the HSI values for *O*. *bartramii*. Furthermore, the environmental data in 2010 were set aside as inputs in the AMM-based and GMM-based HSI models for model testing and validation. The monthly frequencies of fishing effort in 2010 were then overlaid on the predicted HSI maps to help select a better HSI model. The procedure of developing the HSI model for *O*. *bartramii* was shown in Fig 2.

### Variability in the squid habitat under anomalous environmental conditions

In this study, we considered El Niño and La Niña phenomena as the anomalous environmental conditions. The El Niño and La Niña phenomena were defined in terms of the SSTA in the Niño 3.4 region (http://www.noaanews.noaa.gov/stories2005/s2394.htm). El Niño, a phenomenon in the equatorial Pacific Ocean characterized by a positive SST departure from normal values in the Niño 3.4 region greater than or equal in magnitude to 0.5°C, averaged over three consecutive months; and La Niña, a phenomenon in the equatorial Pacific Ocean characterized by a negative SST departure from normal condition in the Niño 3.4 region greater than or equal in magnitude to 0.5°C, averaged over three consecutive months [12].

**Fig 3:**
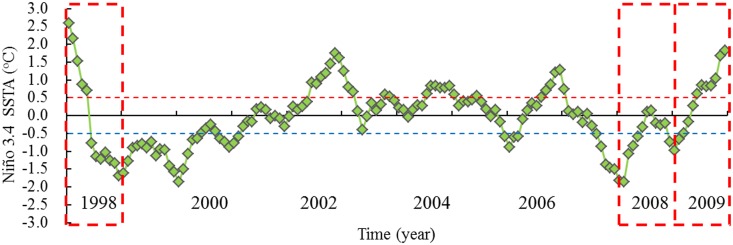
Time series of sea surface temperature anomaly (SSTA) in the Niño 3.4 region. Results indicate that a La Niña event, average environmental condition and an El Niño event corresponded to the fishing season in the years of 1998, 2008 and 2009, respectively.

In order to examine how the oceanographic variability influenced the distribution and abundance of *O*. *bartramii*, we illustrated the habitat use of the species during attempted to example three representative years of contrasting catches, corresponding to a very high (1998), average (2008) and extremely low (2009). Based on the definitions for the El Niño and La Niña, there were two El Niño events (January-May 1998, June-December 2009) and three La Niña events (July-December 1998, January-April 2008 and December 2008-March 2009) in 1998, 2008 and 2009 (Fig 3). It was clearly indicated that the a La Niña event, average environmental condition and an El Niño event corresponded to the periods from July to November in 1998, 2008 and 2009, respectively.

The HSI values under the three different environmental conditions were predicted based on the selected HSI model. The average HSI values on the fishing ground were initially calculated to examine the squid habitat quality for each year. Additionally, we defined the fishing ground with HSI<0.2 as poor squid habitat, whereas the fishing ground with HSI>0.6 as favorable habitat for *O*. *bartramii*. The percentages of the poor habitat and the suitable habitat areas accounting for the total areas of fishing ground were evaluated and compared in the three years. More importantly, the inter-annual environmental change played an important role in regulating the spatio-temporal variability in the suitable habitat and the latitudinal gravitational centers of fishing effort (LATG). In this study, the locations of suitable area of *O*. *bartramii* for each fishing month in 1998, 2008 and 2009 were determined by averaging the latitude of the area with the HSI>0.6 on the fishing ground [43]. The monthly LATG was calculated following the method from Lehodey et al. [44]:

$$LATG_{m}=\frac{\sum \left(Latitude_{i,m}\times Effort_{i,m}\right)}{\sum Effort_{i,m}}$$ (9)

where *Latitude*_*i*,*m*_ denoted the latitude of the *i*th fishing unit of 1°×1° in month *m*;*Effort*_*i*,*m*_ was the total fishing efforts in area *i* in month *m*.

## Results

### The frequency distribution of fishing efforts and optimal range of each environmental variable

**Fig 4:**
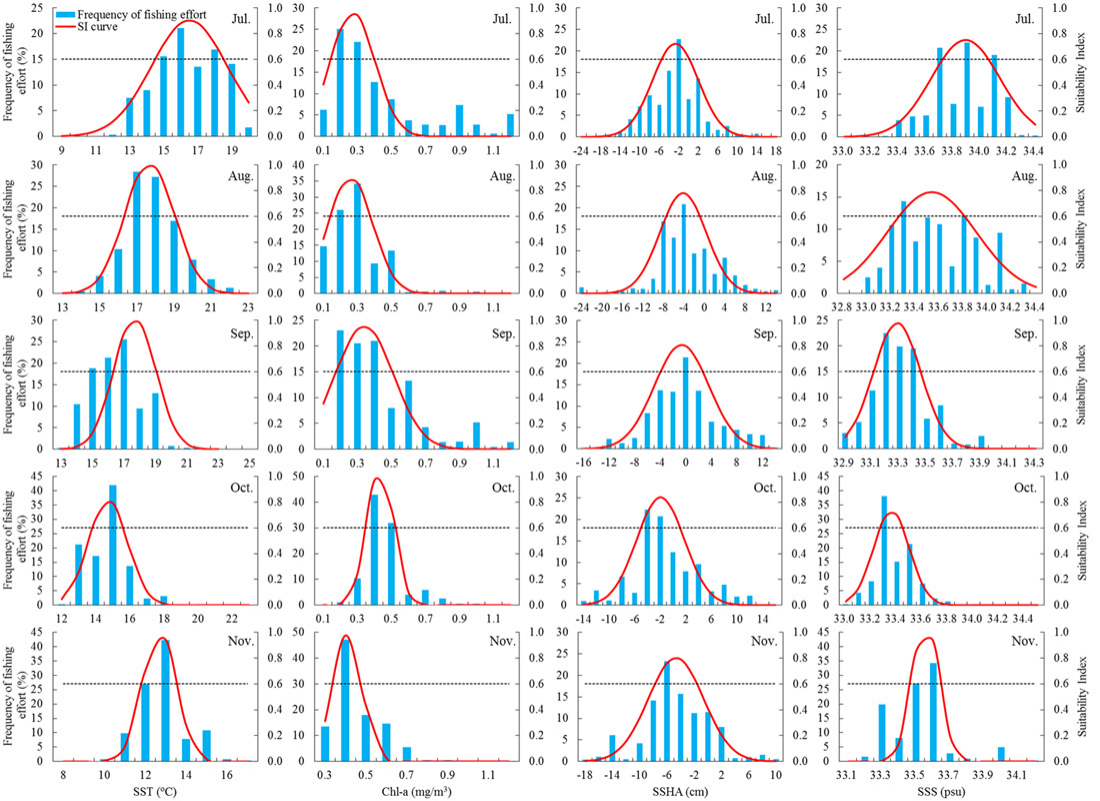
The frequency of fishing effort on each environmental variable and estimated SI curve for each environmental variable on the traditional fishing ground of *Ommastrephes bartramii* during July to November in the Northwest Pacific. The interaction between the SI curve and the dashed line donates the optimal range of each environmental variable (SI = 0.6).

The distribution of fishing effort in relation to the environmental variables varied monthly and interannually (Fig 4). In July, high frequency of fishing efforts were mainly distributed in the waters with SST ranging from 15 to 18°C, Chl-a ranging from 0.2 to 0.3 mg/m^3^, SSHA ranging from -4 to -2 cm, and SSS ranging from 33.7 to 34.1 psu (practical salinity units). During August, the fishing efforts tended to be concentrated in the areas with SST from 17 to 19°C, with Chl-a from 0.2 to 0.3 mg/m^3^, with SSHA from -8 to -4 cm, and with SSS from 33.2 to 33.8 psu, respectively. The fishing efforts in September were mostly taken place on the fishing grounds where the SST varied from 15 to 17°C, Chl-a varied from 0.2 to 0.4 mg/m^3^, SSHA varied from -4 to 2 cm, and SSS varied from 33.2 to 33.4 psu. Furthermore, the preferred ranges with high fishing efforts for each variable in October corresponded to SST between 13 and 15°C, Chl-a between 0.4 and 0.5 mg/m^3^, SSHA between -4 and -2 cm, and SSS between 33.3 and 33.5 psu, respectively. However, high fishing effort frequencies in November mainly occurred within relatively narrow ranges from 12 to 13°C, from 0.4 to 0.5 mg/m^3^, from -8 to -4 cm, from 33.5 to 33.6 psu, for SST, Chl-a, SSHA and SSS, respectively.

The models fitted by the SI values and the class interval values of the environmental variables (SST, Chl-a concentration, SSHA and SSS) documented substantial temporal variability (Table 1 and Fig 4). The statistical analysis of each SI model included in this study was highly significant (*P*<0.001) (Table 1). Regression residuals of each SI model followed normal distribution. Moreover, it was found that the optimal range for each environmental variable varied monthly (Table 2). The inferred optimal range of SST by SI models (SI>0.6) tended to be high in July, August and September, were from 14.5 to 18.5°C, 16.3 to 19.0°C, and 14.7 to 17.9°C, respectively. However, the optimal range of SST for *O*. *bartramii* dropped in October and November and tended to be between 13.8 and 15.6°C and between 11.9 and 13.5°C, respectively. For Chl-a concentration, the optimal range tended to vary but with small variations. During July to September, the preferred range of Chl-a was between 0.1 and 0.4 mg/m^3^, but increased to the range between 0.3 and 0.5 mg/m^3^ during October and November. The optimum SSHA in each month appeared to be similarly lower than 0 cm. With respect to SSS, the optimal range was from 33.7 to 34.1 psu in July, from 33.3 to 33.8 psu in August, from 33.1 to 33.5 psu in September, from 33.3 to 33.4 psu in October and from 33.5 to 33.6 psu in November, respectively.

**Table 1:** Fitted suitable index (SI) models for each environmental variable and their parameters estimation during July to November in the Northwest Pacific Ocean.

Month	SI models	*df*	R^2^	*F*	*P*
July	*SI*_*SST*_ = 0.9042 exp(-0.1013(*X*_*SST*_—16.5088)^2^)	11	0.8872	35.3840	0.0001
	*SI*_*Chl-a*_ = 0.9652 exp(-29.356(*X*_*Chl-a*_—0.2723)^2^)	11	0.7588	14.1593	0.0017
	*SI*_*SSHA*_ = 0.7231 exp(-0.0225(*X*_*SSHA*_ + 2.6909)^2^)	20	0.8333	44.9891	0.0001
	*SI*_*SSS*_ = 0.7518 exp(-7.9062(*X*_*SSS*_—33.8887)^2^)	14	0.6335	10.3710	0.0024
August	*SI*_*SST*_ = 1.03 exp(–0.2873(*X*_*SST*_—17.6881)^2^)	10	0.9770	169.5481	0.0001
	*SI*_*Chl-a*_ = 0.9065 exp(–29.9707(*X*_*Chl-a*_—0.2593)^2^)	11	0.8996	40.3159	0.0001
	*SI*_*SSHA*_ = 0.7813 exp(–0.0243(*X*_*SSHA*_ + 4.1734)^2^)	19	0.7970	33.3653	0.0001
	*SI*_*SSS*_ = 0.7879 exp(–3.6697(*X*_*SSS*_—33.533)^2^)	16	0.6069	10.8067	0.0015
September	*SI*_*SST*_ = 0.9362 exp(–0.1695(*X*_*SST*_—16.355)^2^)	12	0.9092	50.0791	0.0001
	*SI*_*Chl-a*_ = 0.9543 exp(–17.1738(*X*_*Chl-a*_—0.3407)^2^)	11	0.7243	11.8248	0.0030
	*SI*_*SSHA*_ = 0.8116 exp(–0.0275(*X*_*SSHA*_ + 0.6517)^2^)	15	0.8859	50.4779	0.0001
	*SI*_*SSS*_ = 0.9788 exp(–16.5003(*X*_*SSS*_—33.2808)^2^)	14	0.9295	79.0653	0.0001
October	*SI*_*SST*_ = 0.8187 exp(–0.3879(*X*_*SST*_—14.717)^2^)	11	0.8225	20.8476	0.0004
	*SI*_*Chl-a*_ = 1.095 exp(–85.2183(*X*_*Chl-a*_—0.4329)^2^)	11	0.9813	236.6393	0.0001
	*SI*_*SSHA*_ = 0.8397 exp(–0.0357(*X*_*SSHA*_ + 1.9538)^2^)	15	0.7472	19.2145	0.0001
	*SI*_*SSS*_ = 0.734 exp(–25.7483(*X*_*SSS*_—33.3562)^2^)	15	0.7856	23.8141	0.0001
November	*SI*_*SST*_ = 1.0106 exp(–0.7552(*X*_*SST*_—12.697)^2^)	9	0.9125	36.4990	0.0002
	*SI*_*Chl-a*_ = 0.9828 exp(–97.4465(*X*_*Chl-a*_—0.4084)^2^)	9	0.8903	28.4134	0.0004
	*SI*_*SSHA*_ = 0.803 exp(–0.0306(*X*_*SSHA*_ + 4.683)^2^)	14	0.8337	30.0868	0.0001
	*SI*_*SSS*_ = 1.1029 exp(–83.3517(*X*_*SSS*_—33.5553)^2^)	11	0.7124	11.1473	0.0037

**Table 2:** The optimal range of each environmental variable estimated by SI models for *Ommastrephes bartramii* during July to November in the Northwest Pacific.

Environmentalvariable	Month				
	July	August	September	October	November
SST (C)	14.5–18.5	16.3–19.0	14.7–17.9	13.8–15.6	11.9–13.5
Chl-a (mg/m^3^)	0.15–0.39	0.15–0.37	0.18–0.50	0.35–0.51	0.34–0.47
SSHA (cm)	-5.5–0.1	-7.4-(-0.9)	-3.9–2.6	-5.0–1.1	-7.7-(-1.6)
SSS (psu)	33.7–34.1	33.3–33.8	33.1–33.5	33.3–33.4	33.5–33.6

### HSI models comparison, selection and validation

**Fig 5:**
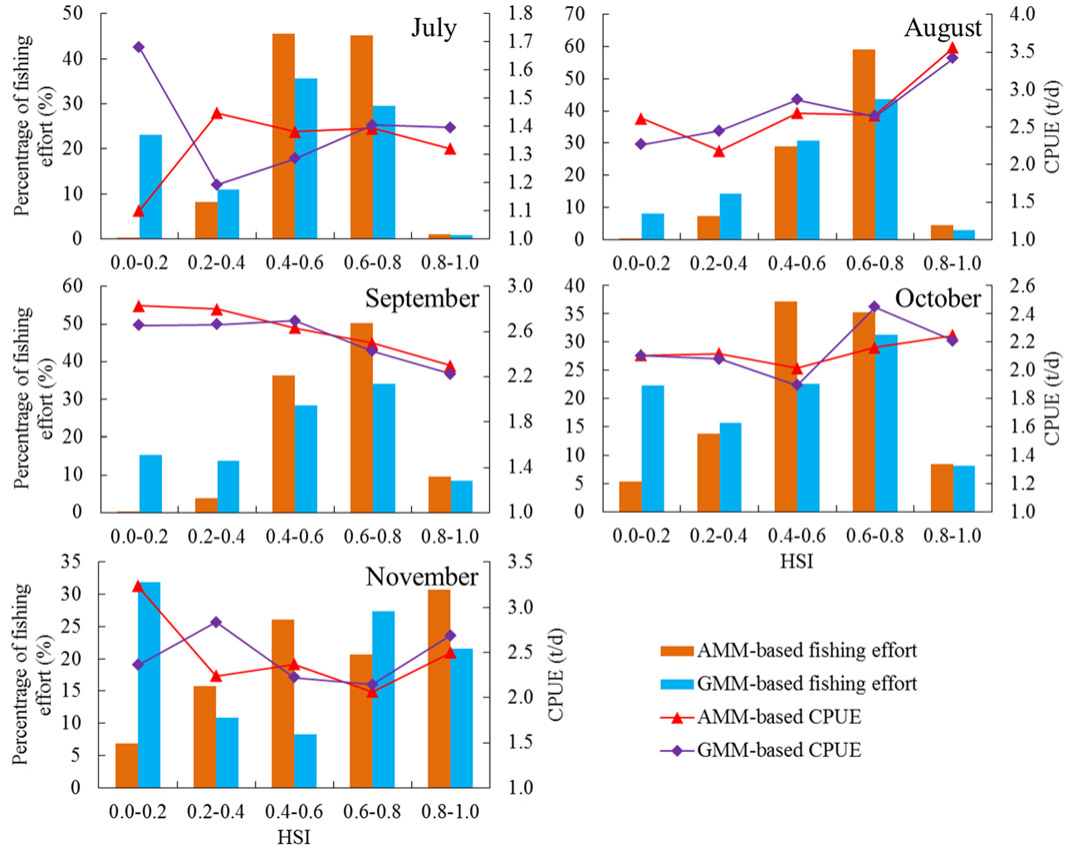
Comparing the performance of arithmetic mean model (AMM) and geometric mean model (GMM). The histogram donates the frequency of fishing effort in each HSI stratum estimated from AMM-based and GMM-based models. The solid line donates the catch per unit effort (CPUE) in each HSI stratum estimated from AMM-based and GMM-based models.

We estimated the average CPUE and percentage of fishing effort within each HSI stratum during 1998 to 2009 and further compared the performances of the two models (AMM and GMM) (Fig 5). High percentages of fishing effort for each month were observed within the AMM-based HSI class range of 0.4–0.6 and 0.6–0.8 and tended to be much higher compared with those within the GMM-based HSI model. For example, the areas with the HSI values of 0.4–0.6 and 0.6–0.8 in July attracted 45.6% and 45.2% of the total fishing efforts, respectively, based on the AMM model. While the fishing sites with the HSI values of 0.4–0.6 and 0.6–0.8 attracted only 35.5% and 29.5% of the total fishing efforts, respectively, according to the GMM model. The fishing efforts were found to be distributed uniformly among the HSI classes for the GMM model. In addition, both models showed that the average CPUE strongly fluctuated, however, the CPUE tended to increase with the HSI values in each month except in September. Clearly, it was notable that the AMM-based model yielded few fishing efforts in the poor habitat with HSI<0.2, and the fishing effort percentages obviously increased with the HSI values, the suitable habitat with the HSI>0.6 accounted for the largest fishing efforts in each month. However, although the majority of fishing efforts occurred in high HSI values based on the GMM-based model, fishing efforts were distributed in poor habitat during July to November. This comparison suggested that the AMM model better captured the theory mentioned in the method section, we thus considered the AMM model to be more suitable than the GMM model.

**Fig 6:**
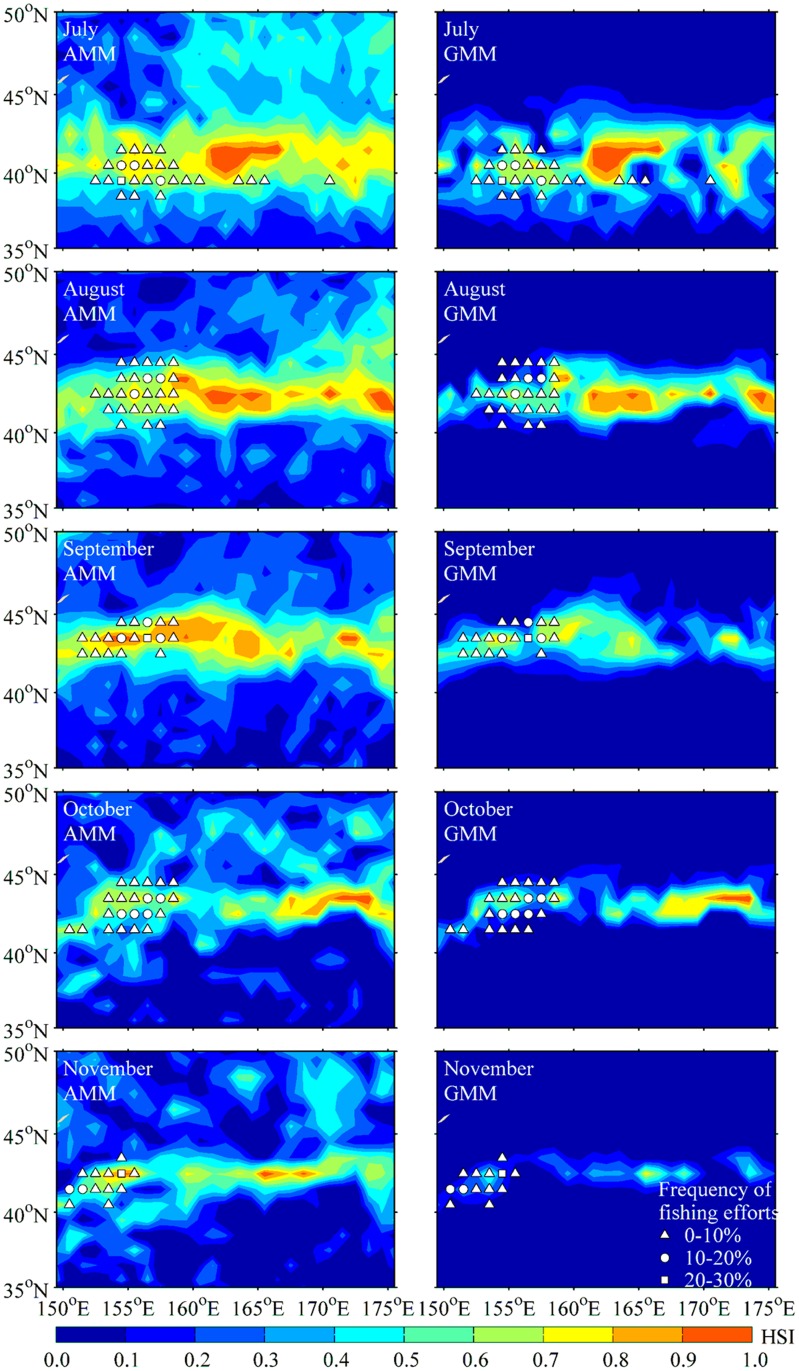
Validation of the AMM-based and GMM-based HSI models. The spatial distribution of the frequency of fishing effort for *Ommastrephes bartramii* in 2010 overlaid on the HSI maps generated from AMM-based model and GMM-based model during July to November.

With the HSI value estimated from the AMM and GMM model during July to November in 2010, the frequencies of fishing efforts were mapped on the predicted HSI maps (Fig 6). The results showed that the suitable areas with HSI>0.6 based on the AMM model were much larger than those estimated by the GMM model. According to the AMM-based HSI model, the fishing effort frequencies tended to increase with the HSI values, the majority of fishing efforts were distributed in the optimal habitats (HSI>0.6). However, the fishing efforts were found to be distributed in the regions with low HSI values predicted by the GMM model in each month, especially in October and November. These findings further indicated that the AMM-based HSI model could provide a more reliable prediction of the suitable squid habitat for *O*. *bartramii* in the Northwest Pacific.

### Variability in the suitable habitat areas under different anomalous environmental conditions

**Fig 7:**
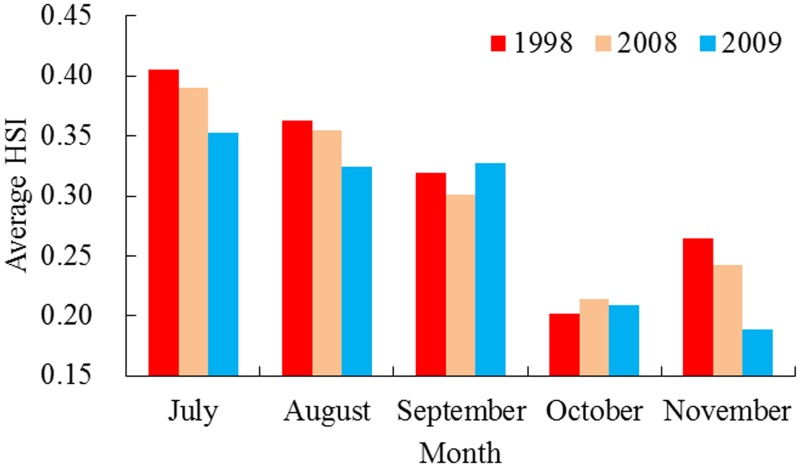
The predicted HSI values for *Ommastrephes bartramii* by the AMM model. Based on the AMM-based model, the HSI value is averaged during July to November in 1998, 2008 and 2009, respectively, on the traditional fishing ground.

The monthly HSI values during July to November in 1998, 2008 and 2009 were predicted based on the AMM model to evaluate the impacts of inter-annual environmental variability on the squid habitat. The average HSI values corresponding to each month from July to November were 0.41, 0.36, 0.32, 0.20 and 0.26 in 1998; 0.38, 0.35, 0.30, 0.21 and 0.24 in 2008; and 0.35, 0.32, 0.33, 0.21 and 0.19 in 2009, respectively (Fig 7).

**Fig 8:**
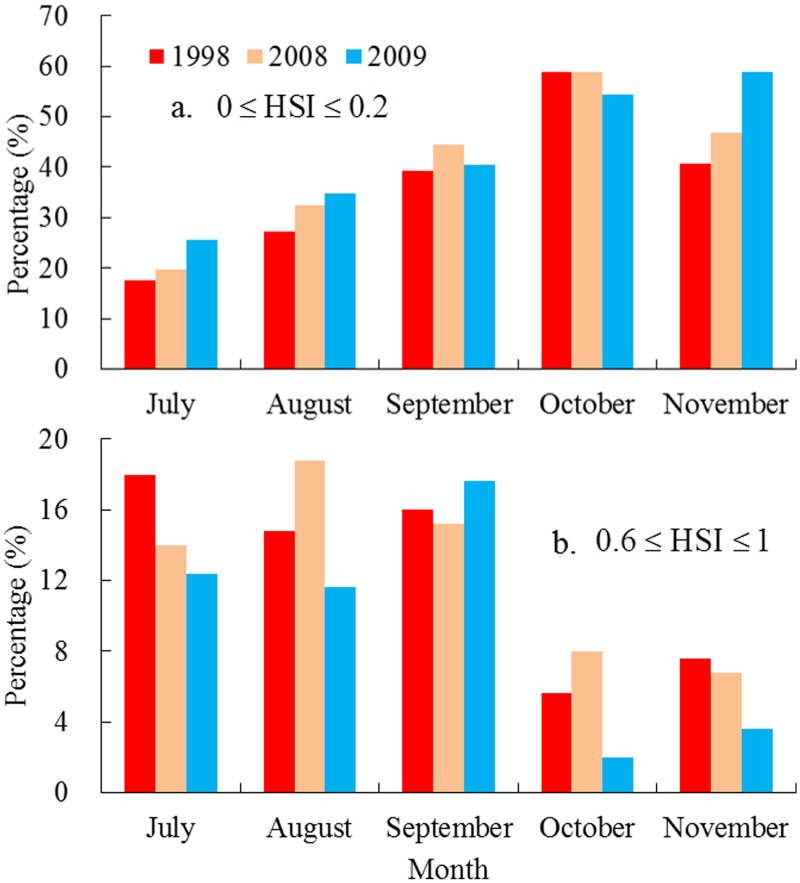
Comparing the different habitat levels during 1998, 2008 and 2009. The percentage of (a) unfavorable habitat area; and (b) suitable habitat area accounting for the traditional fishing ground of *Ommastrephes bartramii* in the Northwest Pacific in 1998, 2008 and 2009, respectively.

The poor squid habitat areas and favorable habitat areas were evaluated under different anomalous environmental conditions. When the fishing ground of *O*. *bartramii* was affected by the La Niña event in 1998, the areas with HSI<0.2 accounted for 17.6% in July, 27.2% in August, 39.2% in September, 58.8% in October, and 40.8% in November of the total area of the traditional fishing ground (Fig 8a); the areas with HSI>0.6 covered approximately 18.0%, 14.8%, 16.0%, 5.6%, and 7.6% of the total fishing ground from July to November, respectively (Fig 8b). The average environmental condition in 2008 yielded 19.6%, 32.4%, 44.4%, 58.8% and 46.8% of the poor habitat areas on the fishing ground during July to November, respectively; while the favorable areas tended to be accounting for a high proportion of the fishing ground over the three years, the values ranged from 6.8% in November to 18.8% in August of the traditional fishing waters. Comparing with those in 1998 and 2008, a significant increase except in October could be seen in the unfavorable habitat areas in each month during 2009, probably as a result of the influence of the El Niño event. The monthly percentage in July to November reached up to 25.6%, 34.8%, 40.4%, 54.4% and 58.8%, respectively. Conversely, the suitable habitat for *O*. *bartramii* in 2009 experienced an obviously decline. The area with the HSI value>0.6 only covered 2.0% of the total fishing ground in October. The largest suitable areas occurred in September, making up 17.6% of the total fishing ground.

**Fig 9:**
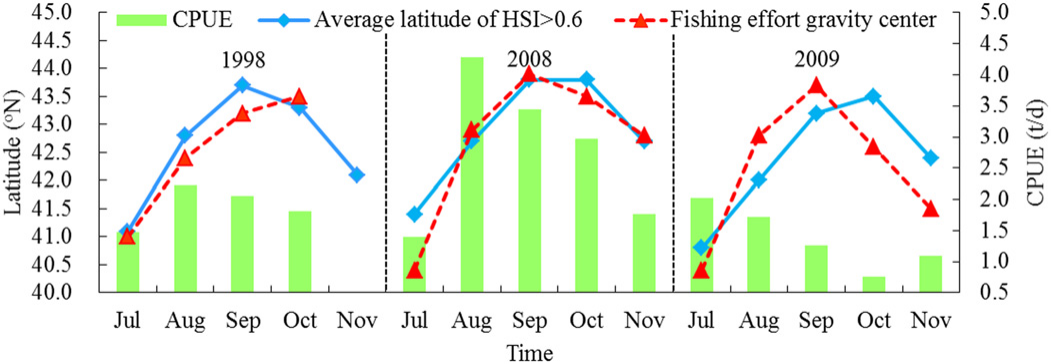
Comparison of the latitudinal gravity center of fishing effort, average latitude of suitable area and CPUE for each fishing month in 1998, 2008 and 2009.

The average latitude of suitable habitat migrated from south to north during July to September, then moved southward during the following fishing months among the three years (Fig 9). The average latitude of suitable habitat corresponding to each month from July to November were 41.1°N, 42.8°N, 43.7°N, 43.3°N and 42.1°N in 1998; 41.4°N, 42.7°N, 43.8°N, 43.8°N and 42.7°N in 2008; and 40.8°N, 42.0°N, 43.2°N, 43.5°N and 42.4°N in 2009, respectively. The estimated monthly LATG in the three years tended to be close to the latitudinal location of suitable habitat but with different distance between them for each month. The LATG in 1998 was 41.0°N, 42.4°N, 43.2°N and 43.5°N, respectively, from July to October; it located at 40.4°N in July, at 42.9°N in August, at 43.9°N in September, at 43.5°N in October and at 42.8°N in November in 2008; however, the LATG moved to the waters distributed at 40.4°N, 42.8°N, 43.7°N, 42.6°N and 41.5°N, respectively, during July to November in 2009. The CPUEs in 1998 tended to be high and fluctuated from 1.48 to 2.23 t/d during July to November. In 2008, the CPUEs were much higher with the values ranging from 1.39 to 4.27 t/d. However, the CPUEs in 2009 fell to 0.76–2.01 t/d.

## Discussion

The HSI modelling approach provided an effective and reliable assessment in evaluating the habitat preference of pelagic species in relation to the relevant environmental variables [45]. This study included the Chinese squid-jigging fishery and environmental data during 1998–2009, this long time-series dataset was used to develop the HSI model, which could truly reflect the habitat characteristics and the preferred habitat for *O*. *bartramii*. The relationship was quantified between the fishing effort and each environmental variable (SST, Chl-a, SSHA and SSS). Significant normal and skewed normal distributions (*P*<0.001) were observed in constructing SI-effort models (Table 1 and Fig 4). The integrated HSI models were further established using the two empirical AMM and GMM models, which were commonly used in developing the HSI model [40–42]. The results derived from the AMM and GMM models showed that the percentage of fishing effort occupied only a small proportion within the HSI value<0.4, the majority of fishing efforts were located in the common (HSI = [0.4 0.6]) and suitable habitats (HSI = [0.6 1.0]) (Fig 5). Therefore, both the models were reliable and basically reflected the distribution of the fishing ground of *O*. *bartramii* in relation to the oceanic conditions. However, the AMM model showed that the percentage of fishing effort increased with the HSI values and little efforts occurred in the poor squid habitat compared to the GMM model results. Thus, the AMM model was considered to be a better choice for the estimation of the HSI values, which was also consistent with previous findings. For example, Chen et al. [34] developed a HSI model to identify the suitable fishing ground for *O*. *bartramii*, they compared the performances of AMM and GMM models by estimating the average CPUE and percentage of fishing efforts according to the grouped HSI values, the results suggested the AMM model was better than the GMM model. Li et al. [43] established a HSI model for chub mackerel (*Scomber japonicus*) in coastal waters of China, they found that the percentages of fishing effort and catch in poor habitat based on the AMM model were much less than those for the GMM model. Consequently, the AMM model was preferred to construct the HSI model to identify the habitat hotspots.

Before we selected the AMM model to predict the HSI values for *O*. *bartramii*, the AMM model was also cross-validated using the environmental data in 2010 (Fig 6). The HSI maps showed a good consistency between the frequency distribution of fishing efforts and the suitable habitat based on the AMM model. The GMM model tended to underestimate the spatial distribution of the suitable habitats for *O*. *bartramii*. The validation results further confirmed the validity of selecting the AMM model as the optimum HSI model.

Previous studies suggested that CPUE was a reliable abundance index to estimate SI values [46]. For example, Chen et al. [47] used the CPUE as the relative abundance index to establish SI curves for chub mackerel in the East China Sea. However, environmental conditions, fishing vessels and technology had great effects on the CPUE, which might introduce biases in developing HSI models. In addition, Tian et al. [28] conducted a study comparing the performance between CPUE-based HSI model and effort-based HSI model for *O*. *bartramii*, and found that the CPUE-based HSI model overestimated the range of suitable squid habitat and underestimated the variations in the spatial distribution of suitable habitat. Therefore, CPUE was not used in the development of HSI models in this study. Fishing effort in the Chinese squid fishery was defined as the integer number of fishing days (each nighttime per one vessel). The fishing effort was a reflection of the density of fishing vessels, the large amounts of fishing efforts implied a good production and high fish abundance [48,49], which could be considered as another index of squid occurrence [34]. The fishing efforts were typically influenced by the environmental conditions and were not randomly distributed, therefore, the HSI modeling would be more objective to explore the suitable habitats for *O*. *bartramii* by using SI-fishing effort models [38].

In the present study, the most suitable habitat for *O*. *bartramii* could be identified by the proxy environmental variables such as SST, Chl-a concentration, SSHA and SSS. These factors indicated the thermal condition, food density and physical features, playing a key role in regulating the squid abundance and distribution [34,49]. We evaluated the optimal ranges of these environmental variables for each month (Table 2). These results in this study were consistent with previous findings [34,50,51]. Within the specific favorable range of the environmental variables, the most productive *O*. *bartramii* habitats could be predicted and detected. In this study, the role of each variable in affecting the dynamics of *O*. *bartramii* stocks was assumed to be equivalent. However, as the different influences of the variables on the distribution of pelagic species, we should carefully differ the roles of each environmental variable in the construction of HSI model. Gong et al. [52] demonstrated that the weighting for different oceanographic variables could greatly affect HSI modelling, different weighting schemes for the relevant variables would result in different spatial distribution of the suitable habitat. Additionally, there had been evidence that the pelagic species responded quickly to the vertical temperature structure [53] and eddy fields [54]. Therefore, the future research should be focused on the development of an integrated HSI model that considers possible differences of environmental variables in influencing the habitat and includes the mixed layer depth and eddy kinetic energy.

*O*. *bartramii* was a short life cycle species, its abundance and distribution were highly vulnerable to anomalous environmental conditions [18]. Previous studies reported that the La Niña and El Niño events strongly affected the squid population dynamics [12,30]. In 1998, 2008 and 2009, the Chinese squid-jigging fishery operated in the similar waters on the traditional fishing ground, the fishing technique and fishing behavior were almost the same. However, 1998, 2008 and 2009 were variable in terms of squid production with typically high, average and low catch, which were in coincidence with the variation in the SSTA in the Niño 3.4 region corresponding to a La Niña event, average environmental condition and an El Niño event, respectively. Great difference in the squid catch and fishing effort distributions over the three years might result from the different environmental conditions. Therefore, the close associations between the fishery production and environmental conditions during the three years were representative, which could be used to evaluate influence of the La Niña and El Niño events on the spatio-temporal dynamics of the squid preferred habitat by HSI modelling approach and to explain the reason caused this change. Exactly, the selection of the three years coincided to the objectives in this study.

Comparing the average HSI values, the poor habitat and favorable habitat areas in 1998, 2008 and 2009 from the outputs of the AMM model, we found that the HSI values experienced pronounced changes under different environmental conditions. High average HSI values occurred in 1998 and 2008, suggesting that the habitat quality for *O*. *bartramii* was at a high level in the two years. While the average HSI value was low in 2009, thereby leading to unfavorable habitat conditions (Fig 7). Moreover, the percentages of areas with HSI values <0.2 in 1998, 2008 and 2009 gradually increased, while the corresponding areas with the HSI value >0.6 gradually decreased (Fig 8). These findings indicated that the favorable habitat greatly expanded in 1998 and abruptly decreased in 2009, implying that the La Niña event in 1998 tended to yield good quality habitat and more favorable regions for *O*. *bartramii* on the traditional fishing ground in the Northwest Pacific, whereas the El Niño event in 2009 yielded an unfavorable habitat condition and less favorable areas for the squid.

**Fig 10:**
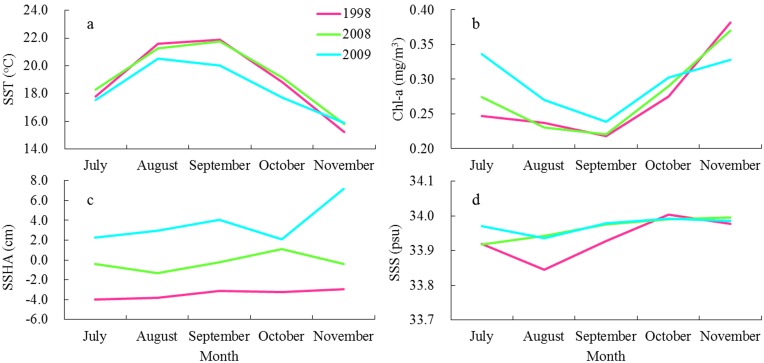
Monthly average environmental variables on the fishing ground of *Ommastrephes bartramii*. Monthly average (a) SST; (b) Chl-a concentration; (c) SSHA; and (d) SSS during July to November in 1998, 2008 and 2009, respectively.

We identified the critical factors that drove the variability in squid habitat and abundance during the years of 1998, 2008, and 2009 corresponding to the La Niña, average and El Niño condition by examining the variations in the environmental variables during the three years (Fig 10). It was of interest that almost identical variations were found in the SST, Chl-a and SSHA in 1998 and 2008. However, apparent anomalous fluctuations occurred in the oceanic conditions on the fishing ground in 2009. The average SST from July to November in 1998 and 2008 tended to be high, but the SSTs throughout the fishing ground in 2009, strongly influenced by the El Niño event, were subject to a large decline. Previous studies suggested that in the suitable temperature range a relatively higher temperature was more favorable for the growth and reproduction of squid [55] and had great impacts on the distribution and the formation of fishing ground [7]. These results suggested that the El Niño event in 2009 yielded an anomalously cool temperature condition on the fishing ground, which was unfavorable for the formation of the suitable squid habitat.

The fishing efforts during July to September were primarily concentrated in the waters with lower Chl-a concentration between 0.2–0.3 mg/m^3^, whereas a large proportion of the fishing efforts were coupled with higher Chl-a concentration about 0.3–0.5 mg/m^3^ in October and November (Fig 4). The suitable ranges of Chl-a concentration for *O*. *bartramii* were low in July to September and high in October and November (Table 2) as well. Surprisingly, the average Chl-a concentration ranging from 0.22 to 0.27 mg/m^3^ during July to September in 1998 and 2008 was lower than that in 2009 which ranged from 0.23 to 0.34 mg/m^3^ on the fishing ground, and the Chl-a concentration was higher in November in 1998 and 2009 comparing to that in 2009 (Fig 10). Ichii et al. [56] suggested that the autumn cohort of neon flying squid preferred to inhabit the waters with the Chl-a of 0.2 mg/m^3^. Fan et al. [57] found that the fishing ground was mainly formed in the area with the Chl-a between 0.10 and 0.30 mg/m^3^. Tang et al. [58] contributed a sharp drop in squid-jigging production in 2009 to the decrease of SST and the high fluctuations of Chl-a concentration. These conclusions all implied that the Chl-a concentration under the La Niña event and average environmental condition in 1998 and 2008 was preferred for the squid, and tended to be a limited factor to the squid abundance in 2009.

Moreover, the SSHA fields tended to be much lower for each month in 1998 and 2008 than those in 2009. The average SSHAs during July to November in 1998 and 2008 were below 0 cm on the fishing ground except in October in 2008. The SSHA in 2009 was relatively high and varied from 2.1 cm to 7.2 cm (Fig 10). According to the optimal range of SSHA for *O*. *bartramii*, the SSHA field in 2009 was unfavorable for the formation of squid fishing grounds. The SSS during the three years did not undergo large fluctuations, indicating that the limited influence of SSS on the squid habitat. Chen and Huang [59] suggested that the distribution of *O*. *bartramii* had strong correlations with SST and Chl-a concentration, but weak with SSS, the results derived in this study tended to be consistent with their findings.

The anomalous environmental variability had potential influences on the latitude of the suitable habitat and the fishing effort gravity centers. Due to the La Niña event in 1998 and intermediate environmental conditions in 2008, the average latitude of the suitable areas tended to move northward than that in 2009 under the El Niño condition (Fig 9). This could partially explain the results from Chen et al. [12] that the fishing ground of *O*. *bartramii* shifted northward in La Niña years and southward in El Niño years. However, we should note that the direct cause-effect links between the environmental change and the squid suitable habitat could not be identified in this study. The squid responded to the yearly changing oceanic conditions probably through changes in physiological and prey conditions. In addition, the strong agreement between the latitudinal variations of suitable habitat and the gravity centers of fishing efforts reinforced the validity of the AMM-based HSI modelling results (Fig 9). The squid abundance was likely associated with the distance between the distribution of productive habitats and the location of the fishing efforts. Between them, the close distance with approximately 0.2–0.3N variations would yield very high abundance (>3.0 t/d), such as the fishing month during August to October in 2008; the intermediate distance with 0.3–0.5N variations might lead to a relatively high squid abundance (1.5–3.0 t/d), such as the fishing dates in August to October in 1998 and July in 2009; a relatively far distance with variations higher than 0.5N consequently resulted in extremely low squid abundance (< 1.0 t/d). It was clearly found that the Chinese squid-jigging vessels in 2009 mostly operated in the waters much northward or southward of the average latitude of suitable habitats on the fishing ground of *O*. *bartramii*, this mismatch at least in part drove the reduction of the catch in 2009.

Through the HSI modelling approach, this study examined the spatio-temporal variability in the suitable habitat for *O*. *bartramii* on the fishing ground in the Northwest Pacific. Our research provided evidence that the strong linkage existed between the anomalous environmental events (ENSO) and the variability in the *O*. *bartramii* habitats. These findings help understand the process of the squid habitat responding to the inter-annual environmental variability. However, further analysis is needed for examining the influences of the anomalous environmental conditions on the dynamics of the squid habitat by improving the HSI models and considering more ENSO events. Overall, our study might provide some potentially valuable insights into exploring the relationship between the underlying squid habitat and inter-annual environmental change.
